# Florida child care center directors’ intention to implement oral health promotion practices in licensed child care centers

**DOI:** 10.1186/s12903-016-0298-5

**Published:** 2016-09-22

**Authors:** Ajay Joshi, Romer Ocanto, Robin J. Jacobs, Vinodh Bhoopathi

**Affiliations:** 1 Pediatric Dentistry Department, Indiana University School of Dentistry, 1121 W. Michigan Street, Indianapolis, IN 46202 USA; 2 Department of Pediatric Dentistry, Nova Southeastern University College of Dental Medicine, 3200 S University Drive, Fort Lauderdale, FL 33328 USA; 3 Psychiatry and Behavioral Medicine, College of Osteopathic Medicine, Nova Southeastern University, 3200 S. University Drive, Fort Lauderdale, FL 33328 USA; 4 Department of Pediatric Dentistry and Community Oral Health Sciences, Temple University Maurice H. Kornberg School of Dentistry, 3223 N Broad Street, Philadelphia, PA 19140 USA

**Keywords:** Child care centers, Day care centers, Oral Health, Oral Health Promotion, Pediatric Oral Health Knowledge, Pediatric Health

## Abstract

**Background:**

To determine the factors associated with child care center directors’ (CCCDs) intention to implement oral health promotion practices (OHPPs) in licensed childcare centers (CCCs) within the next year, and their self-perceived barriers in successfully implementing those practices.

**Methods:**

For this cross-sectional study, a pretested 45-item online survey was sent to 5142 CCCDs assessing pediatric oral health knowledge, attitudes towards oral health, intention to implement OHPPs, and self-perceived barriers to implementing OHPPs. An adjusted logistic regression model determined the factors associated with CCCDs intention to implement OHPPs within the next year.

**Results:**

Participants were 877 CCCDs, with mean age of 48.5 ± 10.5 years, of whom 96 % were women, and 74 % were whites (Response rate = 19.4 %). The majority (67 %) of respondents reported that they intended to implement OHPPs in their center within a year. Insufficient funding, lack of enough training in oral health, and limited time to promote oral health were the most frequently cited barriers to implementing OHPPs. CCCDs of non-White race (*p* = 0.02), with a college degree or above (*p* = 0.05), and with positive attitudes (*p* < 0.0001), were more likely to report that they will implement OHPPs within the next year compared to their counterparts.

**Conclusions:**

CCCDs reported fewer barriers to implementing OHPPs within the next year, indicating that CCCs can be a suitable setting to promote oral health. CCCDs race, educational status and attitudes towards oral health strongly predicted their intention to implement OHPPs. Though this study assessed the intention of CCCDs to implement OHPPs in CCCs, it did not access the actual implementation of OHPPs by them. Therefore future research could longitudinally assess predictors for true implementation of OHPPs. In addition, researchers should adopt a more comprehensive, multi-level approach to assess the actual dental health needs of children attending these centers, along with parental, staff and center level characteristics, and other relevant factors related to implementing OHPPs.

## Background

Dental caries remains a significant health issue among children, with a substantial number of children aged 2 to 15 experiencing dental caries in one or more primary and/or permanent teeth. Data from the 2011–2012 National Health and Nutrition Examination Survey (NHANES), shows that at least 1 in every 5 children aged 2 to 5 years, and approximately 3 in 5 children aged 6 to 8 had dental caries in their primary teeth [1]. Caries prevalence is higher for children aged 12 to 15, with approximately 50 % experiencing dental caries in one or more teeth by that age [1].

The rise of dual working parents, along with single parent households, has led to an increase in the use of out of home child care arrangements in the United States [2, 3]. In 2011, 32.7 million children were in a regular out of home childcare arrangement, out of which, approximately 12.5 million children were 0 to 4 years of age, and 20.2 million children were 5 to 14 years of age [4]. Twenty five percent of these children were in some form of organized facility such as day care or child care centers (CCCs) [4]. In a previous study assessing the dental needs of children in CCCs, approximately 9 % of the children had an urgent need for dental treatment, while almost 2 in 5 children aged 71 months or younger were diagnosed with early childhood caries (ECC) [5]. With substantial numbers of children spending time in organized facilities such as CCCs, specifically at this especially susceptible age range for dental caries, it is expected that the CCCs take adequate measures to counteract this epidemic.

In 2011, the American Academy of Pediatric Dentistry (AAPD) adopted its “Policy on Oral Health in Child Care Centers,” which provides guidance for implementing oral health promotion practices (OHPPs) in out-of-home child care settings [6]. This policy encourages CCCs to implement appropriate OHPPs out of the 14 recommended to reduce a child’s risk of acquiring ECC within their centers. Some of the OHPPs include promoting dental home concept to staff and parents, maintaining dental records for children and an up-to-date emergency and trauma manual, providing optimally fluoridated water to the children, in-service training programs for personnel regarding oral hygiene concepts, proper nutrition choices, minimizing saliva sharing practices, and providing well-balanced and nutrient dense diets.

However, most states’ CCCs regulations insufficiently promote good oral health [7]. One study examined CCCs implementation of major dental health related standards and identified very low oral health content within state regulations specific to dental health prevention [8]. One study that assessed 8 oral health standards (tooth brushing frequency, tooth brush labeling, toothbrush storage, toothbrush availability, tooth brush maintenance, tooth paste availability, oral health screening, and dental contact information) for out of home child care programs across different states identified that the mean number of standards covered per state was only 2.6 out of 8 possible standards [8]. A recent study in Wisconsin declared a need to increase OHPPs in state-funded centers, indicating that OHPPs in CCCs may be inadequate [9].

In general, children in Florida have difficulty accessing dental care, and they may have impending oral health needs [10]. Only 28.6 % of Medicaid-enrolled Floridian children aged 1 to 20 received any dental services in 2012, with only 18.8 and 10.3 % receiving any preventive dental or dental treatment services respectively [11]. With substantial proportions of children spending time away from their parents in some form of organized facility [4], it is logical to assume that there would be benefits to using these non-traditional settings to promote children’s oral health. However, to ascertain whether CCCs are an appropriate setting for oral health promotion and disease prevention, understanding barriers in implementing OHPPs within these centers is pertinent. One factor that may determine OHPPs being implemented within these centers is the willingness of the child care center directors (CCCDs) to do so. CCCDs have a significant role in implementing any health promotion programs within their CCCs. Therefore, this study aims to determine CCCDs intention to implement OHPPs within the next year in licensed Florida CCCs, factors associated with their intention to implement, and various barriers that may impede CCCDs from implementing OHPPs in CCCs.

## Methods

### Design and sample

The Institution Review Board at the Nova Southeastern University Health Professions Division approved this cross-sectional study. The Florida Department of Children and Families maintains an updated, statewide email list of licensed CCCDs, which is publically available and was used for sampling purposes. In January 2014, an email list, although not exhaustive, was available for 5142 licensed Florida CCCDs, and requests for survey participation were made using Survey Monkey® (www.surveymonkey.com). Before administering the 45-item online survey it was pilot tested with 10 CCCDs. Reminders were sent every two weeks after the initial online request, with a total of 3 reminders. The survey was open for completion over a 3-month period (January to March 2014).

### Measures

#### Main independent variables

##### Pediatric Oral Health Knowledge (POHK)

POHK of CCCDs was measured using 3 questions asking about the age when someone should start cleaning a child’s mouth (Correct answer: As soon as tooth erupts), age of first dental visit (correct answer: 1 year), and the most common childhood disease in children (correct answer: tooth decay or cavities). These questions were adapted from a prior study assessing the pediatric oral health knowledge of mothers and guardians [12]. All correct responses were given a score of 1, while incorrect responses were coded as 0. The responses were summed to derive a composite POHK score (Score range: 0 to 3) with higher scores indicating that the POHK of the CCCD was high.

##### Attitudes about Pediatric Oral Health (APOH)

APOH in CCCDs was measured by asking 4 questions that were adapted from a previous study by Mathu-Muju et al. [13]. A 5-point Likert scale (Strongly Agree to Strongly Disagree) was used to rate following statements: 1) cleaning baby teeth is not important, 2) too many other activities to focus on so there is no time for oral health promotion activities, 3) difficult to teach children younger than 3 years old about dental health, and 4) providing oral health promotion activities at the center will prevent tooth decay. The responses from the 4 questions were summed to derive a composite APOH score (Score range: 0 to 20). A higher APOH score indicates positive APOH. The 5-item Likert scales for 4 APOH questions demonstrated an acceptable internal consistency reliability estimate (Cronbach’s alpha = 0.70) with this sample.

##### Self-perceived barriers to implementing OHPPs

To determine the self-perceived barriers that may impede implementing OHPPs within their centers, we asked them to choose from a list of items using a check box option. The respondents were prompted to check all the items that may apply. The researchers developed these items. The list of items (or barriers) included: 1) lack of funding, 2) negative attitudes of parents, 3) cultural or religious beliefs of parents/caregivers, 4) language barriers of parents/caregivers, 5) insufficient oral health training of staff, 6) insufficient space, 7) lack of time, and 8) infection control concerns. We also included an open-ended option allowing CCCDs to indicate additional barriers. All checked responses were coded 1, while non-checked responses were coded 0. A new variable was created and coded 1 for negative responses to the open-ended option, and 0 for no responses. The responses for all items were summed to derive a composite barrier score ranging from 0 to 9, with higher scores indicating higher number of CCCDs self-perceived barriers (and therefore greater difficulty) to implementing OHPPs in CCCs.

#### Main outcome variable

##### Intention to implement OHPPs

The CCCDs were asked if they intend to implement any OHPPs for the children in their center within a year, with a Yes/No response category.

### Analysis

Analyses were performed using SAS statistical analysis software (SAS Institute, Inc. Cary, N.C.) version 9.3. Univariate statistics were utilized for the following main independent variables: CCCD’s age, gender (Male vs females), ethnicity (Hispanics vs Non-Hispanics), race (Whites vs non-Whites), education (College degree and above vs Less than a college degree), annual income (> = $50,000 per year vs < $50,000), CCCDs years of experience working at a CCC, POHL, APOH, and self-perceived barriers. One multivariate adjusted logistic regression model determining CCCDs intention to implement OHPPs within the next year in their CCC was created, adjusting for the above-mentioned independent variables. We performed multicollinearity diagnostic analysis and determined that no collinearity existed between the main independent variables.

## Results

Fifty-three CCCDs opted out of study participation, and 631 email addresses were invalid. Responses from 877 participants were received, yielding an overall response rate of 19.4 % (877/4511). The mean age of the study participants was 48.5 ± 10.5 years.

Table 1 provides an overview of the study sample’s demographic characteristics. The majority of the respondents were women (96 %). Nineteen percent of the respondents self-identified as belonging to the Hispanic ethnicity. The sample was predominantly White (74 %); while Black/African Americans made up 22 % of the sample. The remainder of the sample identified their race as “other” (3 %), Asian (1 %), and Native Hawaiian/Pacific Islander (0.2 %). Sixty-five percent of the respondents reported having earned a college degree or higher. Approximately 61 % reported earning an annual income of less than $50,000.

**Table 1:** Demographic characteristics of participating Florida childcare center directors

Gender	%
Female	96 %
Male	4 %
Ethnicity	
Not Hispanic or Latino	81 %
Hispanic or Latino	19 %
Race	
American Indian or Alaska Native	0 %
Asian	1 %
Black or African American	22 %
Native Hawaiian or Other Pacific Islander	0.2 %
White	74 %
Other	3 %
Highest level of formal education completed	
High school diploma/GED	5 %
Some College	22 %
Vocational/Technical College	8 %
College Degree	46 %
Post-graduate degree	19 %
Income	
0–$15,999	3 %
$16,000–$29,999	20 %
$30,000–$49,999	38 %
$50,000–$69,999	14 %
$70,000 and above	7 %
Prefer not to answer	18 %

Fewer than 20 % of respondents correctly answered “no” when asked if the age to start cleaning a child’s mouth was 1 year, and only 35 % correctly knew that a child’s first dental visit should not be at 2 years (Table 2). However, 84 % of respondents correctly identified tooth decay as the most common childhood disease. The overall mean POHK score was low at 1.3 ± 0.8 (mean ± SD) out of a maximum score of 3.

**Table 2:** Childcare center directors’ oral health knowledge and attitudes

Oral Health Knowledge	
Start cleaning child’s mouth at the age of 1.	%
Yes	82 %
No ^a^	18 %
The first dental visit for a child should be at 2 years.	
True	65 %
False ^a^	35 %
Most common childhood disease in children under 7 years of age	
Asthma	13 %
Hay fever	1 %
Tooth decay or cavities ^a^	84 %
Chicken Pox	2 %
Attitudes	
Cleaning baby teeth is not that important	%
Strongly agree	2 %
Agree	1 %
Not sure	3 %
Disagree	19 %
Strongly disagree	75 %
Too many activities to devote any time to dental health	
Strongly agree	2 %
Agree	7 %
Not sure	14 %
Disagree	42 %
Strongly disagree	35 %
Teaching children younger than 3 years of age about dental health is too difficult	
Strongly agree	2 %
Agree	3 %
Not sure	7 %
Disagree	42 %
Strongly disagree	45 %
Don’t believe the activities provided in the center will prevent cavities	
Strongly agree	3 %
Agree	9 %
Not sure	20 %
Disagree	34 %
Strongly disagree	34 %

^a^Indicate correct answers

The attitudes of CCCDs are described in Table 2. We found that the majority (94 %) of respondents agreed that cleaning baby teeth was important to the overall health of the child. Most disagreed that there were too many activities in CCCs to focus on oral health (77 %), and that teaching children younger than 3 years old about oral health was too difficult (87 %). However, only 68 % of the participants agreed that providing oral health promotion activities in the center would prevent dental caries. The overall mean APOH score for the sample was 16.8 ± 2.7 (mean ± SD) out of a maximum score of 20, indicating positive attitudes towards oral health.

**Fig. 1:**
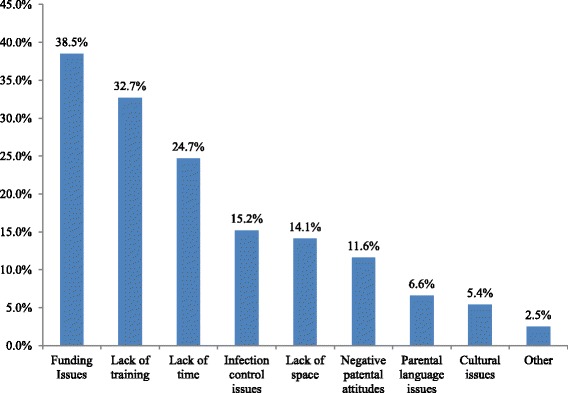
Self-perceived barriers in implementing OHPPs as reported by CCCDs

A majority (67 %) of the respondents reported that they were intending to implement OHPPs for children in their center within the next year. Figure 1 illustrates the frequency of reported barriers they will face in implementing OHPPs within their centers. The most frequently selected self-perceived barrier was financial constraints (38.5 %), followed by insufficient training of the staff to promote oral health (32.7 %) and lack of time (24.7 %). Family/parent’s language (6.6 %) or cultural backgrounds (5.4 %) were the least likely to be perceived as barriers by the CCCDs. The overall mean self-perceived barrier score for the sample was 1.55 ± 1.64 (mean ± SD) out of a maximum score of 9, indicating there were lower self-perceived barriers to implementing OHPPs.

The adjusted logistic regression model determining CCCDs intention to implement OHPPs within the next year in their CCCs included the following variables: CCCD’s age, gender, race, ethnicity, income, education, years of experience as a CCCD, POHK, APOH, and self-perceived barriers (Table 3). Results showed that CCCDs of non-White origin (*p* = 0.02), who had a college degree or above (*p* = 0.05), and those with positive attitudes towards pediatric oral health (*p* < 0.0001) were significantly more likely to report that they intend to implement OHPPs within their CCC in the upcoming year.

**Table 3:** Regression model determining CCCDs intention to implement OHPPs within a year in their center

Variables of interest	Odds ratio	95 % CI	*p*-value
Age (higher number)	0.99	0.97–1.01	0.55
Gender (Males vs females)	1.04	0.35–3.14	0.94
Race (Whites vs Non-whites)	0.59	0.37–0.94	**0.02**
Ethnicity (Hispanics vs Non-Hispanics)	1.31	0.76–2.08	0.36
Income (> = 50,000 vs. <50,000)	0.76	0.49–1.19	0.23
Education (College degreee and above vs < college degree)	1.5	1.04–2.27	**0.05**
Years of experience as CCCD (higher number)	1.01	0.99–1.04	0.3
Pediatric Oral health knowledge (higher number)	1.04	0.81–1.33	0.76
Attitudes towards pediatric oral health (higher number)	1.2	1.08–1.27	**<0.0001**
Self-perceived barriers (higher number)	0.9	0.8–1.01	0.07

Bold faced *p*-values indicate statistical significance

## Discussion

A PEW Centers’ report indicated that Florida’s children have difficulty accessing dental care, and that they may have impending oral health needs [10]. Currently, many children spend a large amount of time away from their parents in some form of child care arrangement. Due to the extended period of time in CCCs, the possibility of using these non-traditional settings to promote children’s oral health would be beneficial. Successfully implemented prevention standards or programs depend on the CCCDs efforts and willingness to promote health of the children attending CCCs. Therefore, our study targeted CCCDs in licensed Florida CCCs to examine whether they intended to implement any OHPPs within the next year, factors associated with implementing OHPPS, and the barriers they foresee that may impede the successful implementation of OHPPs .

Results show that while few self-perceived barriers to promote oral health in CCC were identified, the three most common barriers cited by the directors were institutionally driven. Insufficient funding, lack of enough training, and limited time to promote oral health were the most frequently cited reasons. Evidence shows that these three elements are extremely crucial for promoting any effective health promotion or disease prevention program in a CCC [14]. For example, with adequate funding, time, and training, nutritional professionals successfully taught child care staff and directors adopt and operationalize obesity prevention guidelines, thereby creating a supportive feeding environment [14]. Our findings indicate that until the social/structural barriers (money, training, time) at the institutional level are addressed and resolved, successful implementation of effective oral health promotion/interventions may not be possible.

Multivariate modeling showed that CCCD’s who were willing to implement OHPPs in the next year were more likely to be of non-White race, with a college degree or above, and those with better attitudes about pediatric oral health. It is unclear, why CCCDs who identified as racial minorities were more willing to implement OHPPs within the next year, compared to Whites. The literature shows contradictory findings regarding racial/ethnic minorities adopting preventive behaviors compared to Whites [15–17]. Evidence shows that child care staffs with a formal college education or above provide more sensitive and appropriate care to children with asthma [18]. Similarly, our study found that CCCDs with at least a formal college education were more willing to implement OHPPs compared to their counterparts, indicating that someone with higher educational status may understand the importance of implementing OHPPs. Directors with a college degree and above responded more favorably to implementing OHPPs. Therefore, institutions recruiting CCCDs should hire someone with a college a degree and above. Additional training on the importance of oral health for those with low level of education might improve their intention to implement OHPPs. Participants with more positive attitudes about pediatric oral health reported more OHPS implemented in their centers. Evidence shows that those with positive attitudes about maintaining health tend to adopt healthy behaviors themselves [19].

To our knowledge, this is the first study to assess CCCDs’ willingness to implement OHPPs in licensed CCCs, especially in the State of Florida. This study is not without limitations, such as low survey response rate. Low response rates in survey research can influence the findings, and the generalizability (external validity) of the results. Moreover, caution in data interpretation is warranted when data is self-reported, and not observed. The use of a convenience sample of CCCDs working in licensed CCCs did not allow us to survey non-licensed centers, which would have provided more comprehensive and diverse data. Other CCCs, such as family based childcare homes or faith-based centers that provide childcare on a daily basis were not included in this study. In addition, we did not identify if CCCs were a part of a large company or a franchise where barriers for implementing OHPPs may be different compared to a CCC associated with a small business. Selective participation could have introduced bias, with respondents being more interested in the topic compared to non-respondents, raising the possibility that our results over-estimate the attitudes for all Florida licensed CCCDs. CCCDs intention to implement may not always translate into actual implementation of OHPPs, and because we did not follow this cohort longitudinally, it was not possible to determine if the CCCDs actually implemented any OHPPs within the next year.

Future research could longitudinally assess predictors for actually implementing OHPPs. In this kind of environment, research should highlight whether good intentions and/or low self-perceived barriers actually translate into successful implementation of OHPPs, or whether perceived barriers are the strongest predictor of successful implementation. In addition, researchers should adopt a more comprehensive, multi-level approach to assess the actual dental health needs of children attending these centers, along with parental, staff and center level characteristics, and other relevant factors related to implementing more OHPPs. Such a study should also include all types of CCCs (e.g., family based, faith based, school based) and how these multilevel factors interplay to impact the oral health of children.

## Conclusions

CCCs are an appropriate and innovative setting for dental disease prevention and oral health promotion. Novel programs can be adopted in these centers to prevent dental diseases and promote good oral health behaviors among child enrollees. Implementing simple oral health promotion strategies in CCCs, may have a major impact on the oral health of the children. CCCDs in this study reported low overall oral health knowledge, and therefore, it is dental professionals’ and dental associations’ responsibility to educate the CCCDs, including the staff at the CCC, and the parents of the children to promote good oral hygiene in children.

## Abbreviations

AAPD: American Academy of Pediatric Dentistry
APOH: Attitudes about Pediatric Oral Health
CCCDs: Child care center directors
CCCs: Child care centers
OHPPs: Oral health promotion practices
POHK: Pediatric Oral Health Knowledge
